# Associations of Environmental Exposure to Arsenic, Manganese, Lead, and Cadmium with Alzheimer’s Disease: A Review of Recent Evidence from Mechanistic Studies

**DOI:** 10.3390/jox15020047

**Published:** 2025-03-24

**Authors:** Giasuddin Ahmed, Md. Shiblur Rahaman, Enrique Perez, Khalid M. Khan

**Affiliations:** 1 Department of Biology and Chemistry, Texas A&M International University, Laredo, TX 78041, USA; enriqueperez@dusty.tamiu.edu; 2 Department of Public Health, College of Health Sciences, Sam Houston State University, Huntsville, TX 77341, USA; mxr291@shsu.edu; 3 Department of Environmental Science and Disaster Management, Noakhali Science and Technology University, Noakhali 3814, Bangladesh

**Keywords:** Alzheimer’s disease, neurodegenerative diseases, heavy metals, arsenic, cadmium, mechanistic studies

## Abstract

Numerous epidemiological studies indicate that populations exposed to environmental toxicants such as heavy metals have a higher likelihood of developing Alzheimer’s disease (AD) compared to those unexposed, indicating a potential association between heavy metals exposure and AD. The aim of this review is to summarize contemporary mechanistic research exploring the associations of four important metals, arsenic (As), manganese (Mn), lead (Pb), and cadmium (Cd), with AD and possible pathways, processes, and molecular mechanisms on the basis of data from the most recent mechanistic studies. Primary research publications published during the last decade were identified via a search of the PubMed Database. A thorough literature search and final screening yielded 45 original research articles for this review. Of the 45 research articles, 6 pertain to As, 9 to Mn, 21 to Pb, and 9 to Cd exposures and AD pathobiology. Environmental exposure to these heavy metals induces a wide range of pathological processes that intersect with well-known mechanisms leading to AD, such as oxidative stress, mitochondrial dysfunction, protein aggregation, neuroinflammation, autophagy dysfunction, and tau hyperphosphorylation. While exposure to single metals shares some affected pathways, certain effects are unique to specific metals. For instance, Pb disrupts the blood–brain barrier (BBB) and mitochondrial functions and alters AD-related genes epigenetically. Cd triggers neuronal senescence via p53/p21/Rb. As disrupts nitric oxide (NO) signaling, cortical, and synaptic function. Mn causes glutamate excitotoxicity and dopamine neuron damage. Our review provides a deeper understanding of biological mechanisms showing how metals contribute to AD. Information regarding the potential metal-induced toxicity relevant to AD may help us develop effective therapeutic AD intervention, treatment, and prevention.

## 1. Introduction

Alzheimer’s disease (AD) is one of the most prevalent neurodegenerative diseases across the world [1,2]. About 50 million individuals worldwide suffer from AD or AD-related dementia, making them a pressing global health concern [3,4]. It has been projected that the prevalence of AD dramatically increases due to rising life expectancy worldwide. As the degeneration of neurons takes place, AD gradually impairs cognitive abilities, including memory, attention, language, judgment, and thinking [5]. In the advanced stages of AD, patients experience significant memory loss, confusion, hallucinations, and a lack of self-sufficiency. Eventually, AD patients die from respiratory syndrome, infection, or starvation [4]. Additionally, AD is the most common form of dementia among older adults [6]. The increase of AD and Alzheimer’s disease related dementia (ADRD) to an estimated 152 million people by 2050 will put remarkable pressure on healthcare systems [7,8]. The initial stage of AD is characterized by the aggregation of amyloid β-protein (Aβ) in senile plaques, the formation of neurofibrillary tangles composed of abnormally phosphorylated tau protein, and a reduction in neuronal cell number, all contributing to declining cognition [6,9]. Currently, four genes are known to contribute to the development of AD: (i) the amyloid precursor protein (APP), (ii) presenilin 1 (PSEN1), (iii) presenilin 2 (PSEN2), and (iv) the apolipoprotein E (APOE) gene [10,11].

The typical onset of sporadic or late-onset AD may be associated with several environmental, lifestyle, and genetic risk factors and thus considered as a multifactorial disorder [12,13,14,15,16,17]. The most studied mechanisms of the AD pathogenesis are Aβ protein and tau-associated mechanisms [18,19], glial dysfunction [20], mitochondrial dysfunction [21], oxidative stress [22,23], neuroinflammation [24], calcium dysregulation [25,26], as well as various lifestyle and environmental factors also associated with AD pathobiology [4,12,27]. Although age, genetics, and lifestyle factors have been established as primary risk factors to the onset of AD, growing evidence indicates that environmental exposures, such as toxic metals, may have adverse effects on AD/ADRD pathogenesis [16,28,29,30].

Among the environmental risk factors for AD and ADRD, air pollution including exposure to ozone, heavy metals, pesticides, Bisphenol A, and microplastics, are the most prominent risk factors [29]. These factors may be associated with oxidative stress or inflammation as well as numerous genetic and environmental risk factors that intersect with the etiology of AD and, therefore, illustrate the complexity of the illness [31]. Experts have proposed multiple mechanisms to elucidate the complexity of AD. For instance, Henderson et al. (1988) proposed two hypotheses based on epidemiological data [32]. The first hypothesis suggests that toxic or pathogenic particles aggregate in the brain and may induce AD over time. The second hypothesis suggests that AD develops due to a combination of environmental exposure, genetic susceptibility, and age-related biological changes. These factors collectively contribute to cerebral and neuronal dysfunction, ultimately leading to AD [29,32]. Environmental exposure to metals may induce AD or ADRD through one of the two mechanisms highlighted in the above-mentioned hypotheses.

Environmental toxicants, especially heavy metals including arsenic (As), manganese (Mn), lead (Pb), and cadmium (Cd), are widespread pollutants that accumulate in various ecosystems due to human activities such as industrialization, mining, agricultural practices, and urbanization [33,34]. Studies have found that environmental exposure to heavy metals may induce neurotoxicity and possibly influence the onset of neurodegenerative disorders such as AD and Parkinson’s disease (PD) [1,35,36,37]. Prospective associations between neurodegenerative disease (i.e., AD) and elevated exposure to specific metals (such as As, Mn, Pb, and Cd) have been found through epidemiological and experimental investigations [35,38,39,40,41,42,43,44]. For example, a population-based cross-sectional study demonstrated that prolonged As exposure may adversely affect adults’ cognitive performance in a dose-dependent manner [42]. Likewise, Mn, a necessary trace element, has shown neurotoxic effects at high concentrations that may cause damage to dopaminergic neurons and cognitive impairment leading to AD development [45,46,47]. A study was conducted among Chinese elders by Tong et al. and found Mn levels markedly elevated in the brains of AD patients with dementia in comparison to healthy individuals, suggesting that high Mn levels may have a role in the progression of AD as a critical pathogenic factor [48]. Prolonged Pb exposure is historically linked to cognitive deficit in children [49,50]. Exposure to Pb during childhood has also been associated with an increased risk of developing neurodegenerative disorders, including AD in adults [51,52]. In the context of neurodegeneration, Cd exposure is involved in calcium signaling disruption [53], inducing oxidative stress [54], and causing mitochondrial dysfunction [55], which are intricately associated with AD pathology [56,57]. A population-based study conducted by Peng and colleagues demonstrated a positive relationship between Cd exposure and mortality from AD [58]. Similarly, another study showed a significant association between blood Cd levels and AD mortality among older adults in the USA [39].

Despite it being over a century since AD was first identified by German psychiatrist and neuropathologist Dr. Alois Alzheimer in 1906, intricate molecular pathways underlying the pathophysiology of AD remain inadequately understood [9,59]. In particular, the development of AD induced by metal exposure primarily due to As, Mn, Pb, and Cd and the underlying mechanisms are yet to be fully understood. In the current review, we have focused on As, Mn, Pb, and Cd as they are more commonly studied heavy metals and potential neurotoxicants. Moreover, they can directly harm the nervous system and cause neurological damage in individuals after exposure [60,61,62]. Notably, most published reviews have focused on single metal exposure rather than providing comprehensive insights and thorough comparisons of the mechanisms that illustrate the pathways of the development of AD/ADRD [2,63,64,65,66]. Therefore, the aim of this review is to summarize contemporary mechanistic research exploring the links between multiple metal exposures, such as As, Mn, Pb, and Cd, and their contribution in AD development using data from the most recent mechanistic studies (i.e., studies conducted in the past 10 years). Through the review of the recent data, we seek to pinpoint common routes, processes, and molecular mechanisms by which these metals could affect and/or contribute to the development and onset of AD.

## 2. Methods

### 2.1. Search Strategy

**Figure 1:**
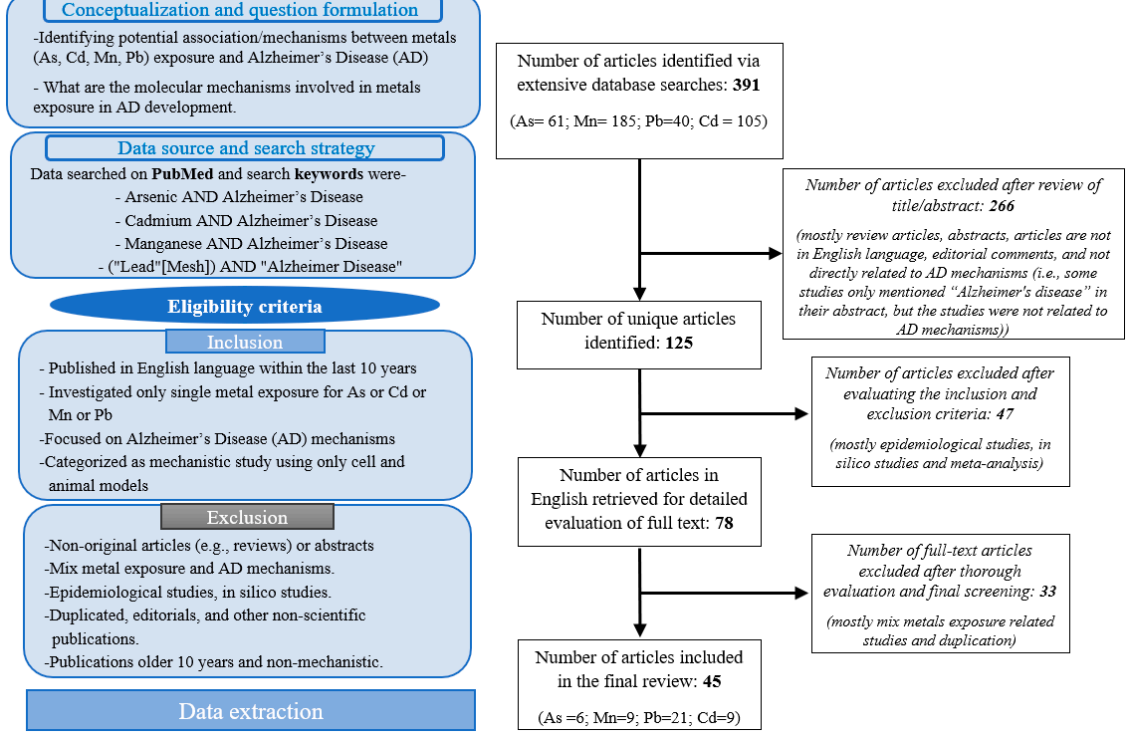
Details of data source, search strategy, eligibility criteria, and study selection process.

Relevant primary research articles, published from 2014 through 2024, were identified using the PubMed Database. The literature search was conducted during August–September 2024 to collect information on the associations of As, Mn, Pb and Cd with Alzheimer’s disease (AD). We restricted our searches to studies published in peer-reviewed English-language journals, within the last 10 years. The following keywords were used: Arsenic AND Alzheimer’s Disease; Cadmium AND Alzheimer’s Disease; Manganese AND Alzheimer’s Disease; and (“Lead” [Mesh]) AND “Alzheimer Disease” [Mesh]. A total of 391 articles were retrieved and screened using the following inclusion and exclusion criteria (Figure 1). Selected articles were then retrieved for full-text screening and data extraction.

### 2.2. Inclusion Criteria

Studies were included if (1) published in the English language within the last 10 years, (2) they investigated only single metal exposure for As or Cd or Mn or Pb, (3) they focused on Alzheimer’s disease (AD) mechanisms, and (4) they were categorized as mechanistic study using only cell and animal models (Figure 1).

### 2.3. Exclusion Criterion

Studies were excluded if they (1) did not focus on AD mechanisms, (2) were non-original articles (e.g., reviews) or abstracts, (3) investigated mixed metal exposure and AD mechanisms, (4) were epidemiological studies, in silico studies, (5) were duplicated, editorials, and other non-scientific publications, or (6) were older than 10 years or non-mechanistic (Figure 1).

### 2.4. Data Extraction

The following predetermined variables were extracted from the selected articles (*n* = 45): (1) bibliographical data (authors name, year of publication), (2) objective of the study, (3) design of the study, (4) exposure, and (5) key findings of the study. A total of 125 unique studies were identified. After reviewing abstracts, full-text articles, and their reference sections, 45 articles were finally selected for further analysis for this review (Figure 1).

## 3. Results

After an extensive literature search and final screening, a total of 45 original research articles were selected for this review. Among the 45 articles, 6 are related to As exposure, 9 to Mn exposure, 21 to Pb exposure and 9 to Cd exposure and AD pathobiology. The results of the selected studies are presented in Table 1, Table 2, Table 3 and Table 4.

### 3.1. Mechanisms and Pathobiology of Alzheimer’s Disease Development Related to Arsenic Exposure

Arsenic (As) is one of the well-known naturally occurring toxic metalloids prevalent in the Earth’s crust. Drinking water and As-contaminated foods are the primary sources of human exposure to environmental As [67,68]. Although the molecular mechanism of As-associated pathobiology in Alzhemer’s disease (AD) remains unclear, experimental research and epidemiological data indicate that As produces neurotoxicity and affects memory and cognition, which are the symptoms of the AD patients [2,69]. Exposure to environmental As and its metabolites triggers several pathogenic events, including oxidative damage, inflammation, mitochondrial dysfunction, ER stress, apoptosis, compromised protein homeostasis, and aberrant calcium signaling. Research has shown that these changes align with the majority of AD’s pathological, biochemical, and clinical manifestations [2].

This review provides updated mechanistic insights into the impact of As exposure on AD development and pathology, based on six recent studies. Of these, three were conducted using mice models [41,70,71], two were conducted on rats [72,73], and one study was carried out using SH-SY5Y cells [74]. The key findings from these mechanistic studies are grouped into categories, which are discussed below and summarized in Table 1.

**Table 1:** Literature summary of the mechanistic studies for arsenic (As) exposure and AD pathobiology.

SL	References	Study Objective	Study Type	Exposure	Key Findings
1	Tripathi et al., 2022 [41]	Investigated nitric oxide signaling in As neurotoxicity using mice model.	Mice model	Mice—drinking water (0.1 ppm and 1 ppm for one month)	As disrupts nitric oxide (NO) regulation, antioxidant defense, and S-nitrosylation (SNO) signaling in the striatum and hippocampus, resembling features of ASD and AD pathobiology.
2	Niño et al., 2021 [72]	Investigated synaptic structure (cortical microstructure and synapses) in chronic As exposure using both a triple-transgenic AD model and Wistar rats.	Triple-transgenic AD model and Wistar rats	Rats—drinking water (3 ppm; until 2 and 4 months of age)	As exposure changes cortical microstructure, decreases synaptic connectivity, increases spine density as well as causes structural changes in the cortex and synaptic regions.
3	Wisessaowapak et al., 2021 [74]	Investigated if prolonged exposure to As affected the phosphorylation of wild-type tau in the neuronal cell model (SH-SY5Y Cells).	SH-SY5Y Cells	Cells (1, 5, or 10 μM for 1, 3, or 5 days)	As induces tau hyperphosphorylation, mediated by GSK3β and ERK1/2 activation, which may contribute to the development of sporadic AD.
4	Pakzad et al., 2021 [70]	Investigated the correlation between arsenic trioxide exposure and its impact on the tau protein Ser262 phosphorylation in male mice.	Mice model	Mice—drinking water (1 and 10 ppm for 3 months)	As exposure increases tau phosphorylation at Ser262.
5	Niño et al., 2018a [71]	Investigated As exposure and the pathophysiological progress of AD using the 3xTgAD mouse model.	3xTgAD mouse model	Mice—drinking water (3 ppm; until 6 months of age)	As exposure accelerates neurodegenerative processes via mitochondrial dysfunction (bioenergetic dysfunction) and increases oxidative stress, key factors to AD pathology.
6	Niño et al., 2018b [73]	Investigated the effects of chronic As exposure on the production and elimination of Amyloid-β (Aβ) in Wistar rats.	Male Wistar rats	Rats—drinking water (3 ppm; until 4 months of age)	As exposure causes behavioral deficits and increases in cleaved Aβ (1–42) levels in brain lysates, potentially disrupting Aβ processing and promoting amyloid accumulation in the brain, hallmark of AD.

#### 3.1.1. Arsenic and Bioenergetic Dysfunction in Alzheimer’s Disease

Bioenergetic dysfunction is a hallmark in Alzheimer’s disease (AD) and plays a crucial role in its pathogenesis. Employing the 3xTg-AD mouse, a well-established and widely used model for AD, Nino et al. demonstrated that chronic As exposure (3 ppm) from gestation to 6 months disrupts mitochondrial bioenergetics. Mitochondria isolated from the hippocampus of these mice exhibit reduced ATP production, impaired respiratory chain activity, and increased reactive oxygen species (ROS) generation. Immunohistochemical analysis further showed increased amyloid plaques and tau phosphorylation, two characteristic features of AD pathology. Behavioral studies revealed spatial memory deficits and disruption of circadian rhythms, indicating that arsenic accelerates neurodegenerative processes through mitochondrial dysfunction and exacerbating oxidative stress, both of which are central to AD pathology [71].

#### 3.1.2. Arsenic and Tau Phosphorylation: A Key Marker of Alzheimer’s Disease

The increased level of hyperphosphorylated tau is another common feature of Alzheimer’s disease (AD). Using male mice, Pakzad et al. explored the effects of different dosages (1 and 10 ppm) of arsenic trioxide exposure on tau protein phosphorylation, specifically at the Ser262 site, which plays a critical role in tauopathies. The researchers found that after three months of exposure to As at 10 ppm, significant increases in tau phosphorylation at Ser262 were observed. Interestingly, low-dose exposure (1 ppm) did not cause significant changes, suggesting a dose-dependent effect of As on tau hyperphosphorylation. The increased tau phosphorylation observed in the high-exposure group parallels key features of AD and tauopathies, reinforcing the idea that As exposure could contribute to the risk of developing neurodegenerative disorders through tau-related mechanisms [70].

#### 3.1.3. Arsenic-Induced Nitric Oxide Dysregulation and Neurotoxicity

Beyond mitochondrial dysfunction and tau pathology, another critical signaling pathway affected by As is nitric oxide (NO) signaling. NO is involved in a variety of cellular processes, including the regulation of mitochondrial function and apoptosis. In an elegant study, Tripathi et al. revealed that As-exposed mice exhibited significant alterations in S-nitrosylation (SNO) signaling pathways, which regulate mitochondrial respiration, antioxidant defense, and apoptosis. These mice also displayed cognitive dysfunctions similar to those observed in autism spectrum disorder (ASD) and AD models. Furthermore, SNO-enrichment analysis revealed disrupted processes involving mitochondrial function, cytoskeleton maintenance, and transcriptional regulation, all of which are critical for neuronal health and survival. These findings underscore the potential role of NO and SNO signaling in As-mediated neurotoxicity and highlight the convergence of As exposure effects with the genetic mutations associated with ASD and AD [41].

#### 3.1.4. Neurotoxicity and Amyloid-β Production, Synaptic, and Cortical Changes

Chronic As exposure is increasingly recognized as a potential environmental risk factor for neurodegenerative diseases such as AD. Several studies have investigated its impact on various cellular and molecular processes associated with neurodegeneration. One study by Niño et al. (2018) investigated the effects of As exposure on amyloid-β (Aβ) production and clearance in Wistar rats. Rats were exposed to 3 ppm of As from gestation to four months of age, resulting in behavioral deficits and a marked increase in cleaved Aβ (1–42) levels in brain lysates, a key pathological feature of AD. This was accompanied by elevated receptor for advanced glycation end products (RAGE) and increased β-secretase (BACE1) activity, suggesting that As exposure may disrupt Aβ processing and contribute to amyloid accumulation in the brain [73].

Building on these findings, a separate study by Niño et al. (2021) examined the effects of chronic As exposure on cortical synaptic connectivity and structure in both Wistar rats and a triple-transgenic AD mouse model. Chronic exposure to 3 ppm sodium arsenite from gestation through postnatal development led to changes in cortical microstructure, as indicated by diffusion-weighted imaging showing altered apparent diffusion coefficient (ADC) and fractional anisotropy (FA) values. Furthermore, the analysis of synaptic markers revealed decreased synaptic connectivity, with abnormal dendritic spine morphology and increased spine density observed at younger ages. These structural changes in the cortex and synaptic regions may underlie As-induced cognitive impairments, which could potentially progress to neurodegeneration in older animals [72].

#### 3.1.5. Tau Hyperphosphorylation and Aggregation

In a recent study, Wisessaowapak et al. (2021) explored the effects of long As exposure on tau phosphorylation in differentiated SH-SY5Y neuroblastoma cells, which may contribute to Alzheimer’s disease (AD) pathogenesis. They showed that low micromolar concentrations of sodium arsenite increased tau phosphorylation at specific residues (pS202), while decreasing dephosphorylated tau (Tau 1). As exposure activated two key kinases: GSK3β and ERK1/2. Inhibition of these kinases using specific inhibitors (BIO, SB216763, lithium, and U0126) reversed the effects of As on tau phosphorylation. Additionally, As exposure increased tau aggregation and promoted the redistribution of tau from the membrane fraction to the cytosol, where neurofibrillary tangles may form. This study suggests that As-induced tau hyperphosphorylation, mediated by GSK3β and ERK1/2 activation, could contribute to the development of sporadic AD [74].

Collectively, the recent mechanistic studies revealed the deleterious impact of As exposure on multiple biological processes of the nervous system, leading to cognitive impairments resembling those observed in AD.

### 3.2. Mechanisms and Pathobiology of Alzheimer’s Disease Development Related to Manganese Exposure

Manganese (Mn) is one of the essential trace metals primarily obtained through dietary sources. It plays a vital role in enzyme function, bone formation, carbohydrate metabolism, and antioxidant activity. While Mn is necessary in trace amounts, inhaling high concentrations of this metal can cause Mn buildup in the brain and manganism, an illness that resembles Parkinsonian disease. Mn accumulation can occur in the brain through dietary intake as it crosses the blood brain barrier (BBB) as well as through inhaled Mn, absorbed by the olfactory transport system. Animal studies have established that excessive Mn exposure can cause oxidative stress, impaired MnSOD activity, and contribute to AD pathology including Aβ accumulation and tau phosphorylation [35,75]. Manganism is a neurological condition caused by chronic exposure to extremely high levels of Mn, often seen in occupational settings like mining, and it manifests with symptoms of cognitive impairments, including motor disturbances like tremors and difficulty walking, often resembling Parkinson’s disease [76]. The effects of Mn exposure on the nervous system are significantly influenced by the levels of other essential metals, particularly iron, as they share similar transport mechanisms in the body. A deficiency in one can increase the uptake of the other, potentially leading to Mn toxicity even at moderate exposure levels [77].

In this review, we summarized findings from nine mechanistic studies that highlight the pathobiology and disease development of AD related to Mn exposure. Five of these studies examined the pathobiology of AD using cell lines [75,78,79,80,81], while the remaining four studies investigated the disease mechanism using both mouse models and cell lines exposed to Mn [40,82,83,84]. The key findings and details of the nine mechanistic studies related to Mn exposure in AD development mechanisms are discussed below and summarized in Table 2.

#### 3.2.1. Drp1 Role in Neuroprotection and Manganese-Induced Toxicity

Dynamin-related protein 1 (Drp1) is crucial for mitochondrial dynamics and fission. Partial inhibition of Drp1 is protective in neurodegenerative disorders like Parkinson’s (PD) and Alzheimer’s (AD), primarily by improving mitochondrial function. However, studies reveal that Drp1 inhibition also reduces protein aggregation, implicating autophagy. Dose-response studies in HeLa and N27 rat immortalized dopamine neurons showed that Mn impairs autophagy flux without affecting mitochondrial function. Autophagy dysregulation was observed in dopamine neurons, but not in GABA neurons of the low Mn-treated mouse. Drp1 knockdown or partial inhibition mitigated Mn-induced autophagy impairment and reduced α-synuclein aggregation, suggesting Drp1′s protective effects extend beyond mitochondrial fission. Drp1 inhibition improves autophagy flux, independent of its mitochondrial role, offering potential therapeutic insights for neurodegenerative diseases linked to autophagy impairment [82].

#### 3.2.2. Impact of Manganese Exposure on Autophagy

Using the mouse N2a blastoma cell line, Xu et al. revealed that Mn levels above 500 μmol/L initiated cell damage and oxidative stress by downregulating PP2Ac methylation, a key regulator of autophagy. This led to mTORC1 activation and autophagy dysfunction. Interventions with the PPME-1 inhibitor ABL-127 and LCMT1 overexpression restored PP2Ac methylation, ameliorating autophagy dysfunction, oxidative stress, and cytotoxicity, suggesting that enhancing PP2Ac methylation offers a protective strategy against Mn-induced neurotoxicity [78].

#### 3.2.3. Manganese Exposure Impairs Astrocytic Glutamate Transporter, EAAT2

Chronic manganese (Mn) exposure impairs the astrocytic glutamate transporter EAAT2, contributing to neurodegenerative diseases like Parkinson’s and Alzheimer’s. Rizor et al. showed that when the H4 human astrocyte cell line is exposed to pathologically relevant concentrations of Mn (250 μM), it induces oxidative stress and TNF-α production, leading to the activation of the IKK-β kinase. This triggers NF-κB p65 translocation, increasing transcription factor Ying Yang (YY1) levels and repressing EAAT2 [75].

#### 3.2.4. REST Protects Dopaminergic Neurons Against Manganese-Induced Neurotoxicity

Chronic manganese (Mn) exposure causes dopaminergic dysfunction and neurodegeneration that result in disorders like manganism and AD. In a recent study, Pajarillo et al. demonstrated that the transcription factor REST represses Mn-induced toxicity in dopaminergic neurons through a concurrent induction of the expression of tyrosine hydroxylase, the enzyme that catalyzes dopamine synthesis. REST binds to the promoter of TH, recruits the epigenetic modifiers CBP/p300, and thereby increases TH transcription, mRNA, and protein. Moreover, REST reduced Mn-induced oxidative stress, inflammation, and apoptosis by reducing TNF-α, IL-1β, IL-6, IFN-γ, and the proapoptotic proteins Bax and Daxx, while promoting antioxidant proteins such as catalase, Nrf2, and HO-1. These results pointed to the potential therapeutic role of REST in treatment against Mn-induced dopaminergic neurodegeneration [79].

#### 3.2.5. Glutamate Excitotoxicity in Manganese-Induced Neurotoxicity

Excessive glutamate stress is implicated in conditions like cerebral ischemia, brain trauma, and neurodegenerative diseases such as PD and AD. Disruption of glutamate homeostasis is central to Mn neurotoxicity. Astrocytes, which maintain glutamate balance and constitute ~50% of CNS neuronal cells, are highly sensitive to Mn-induced toxicity. Their gap junctions (GJ), primarily formed by connexin43 (Cx43), mediate intercellular communication of small molecules like glutamate. In experiments with cultured astrocytes exposed to Mn (0–1000 μM), Lu et al. recently revealed that Mn exposure reduces astrocyte viability, increases apoptosis, and elevates intracellular and extracellular glutamate levels in a dose-dependent manner. Additionally, Mn impairs gap junction intercellular communication (GJIC) by reducing connexin43 (Cx43) expression and inhibiting gap junction function, disrupting the transfer of signaling molecules like glutamate. These findings suggest that Mn-induced glutamate excitotoxicity is driven by GJ dysfunction and reduced Cx43 expression [80].

#### 3.2.6. Role of Manganese Exposure and NF-κB in Neuroinflammation

Microglia are the immune cells of the central nervous system (CNS). They become activated in response to injury, infection, or neurological diseases, including AD, often serving as an early indicator of neurological distress—a process known as microgliosis. Microgliosis is often followed by astrogliosis—reactive responses of astrocytes (another type of glial cell)—both of which contribute to neuroinflammation and exacerbate neuronal damage. While both microgliosis and astrogliosis occur in AD and Mn-induced neurotoxicity, how these glial populations interact with each other is still poorly understood. Kirkley et al. showed that Mn exposure caused dose-dependent increases in pro-inflammatory gene expression and morphological changes in microglia, leading to the release of cytokines and chemokines. Conditioned media from Mn-activated microglia amplified inflammatory responses in astrocytes by increasing mRNA levels of Tnf, IL-1β, IL-6, Ccl2, and Ccl5. Blocking NF-κB inhibited astrocyte activation, while TNF knockdown partially reduced inflammation, highlighting TNF’s role in microglia–astrocyte crosstalk. The findings underscore NF-κB as a key regulator of neuroinflammation and a potential target for reducing astrocytic inflammatory responses and neurodegeneration [81].

#### 3.2.7. Manganese-Induced Toxicity Impairs Glutamatergic Signaling

Manganese (Mn)-induced neurotoxicity and Alzheimer’s disease (AD) exhibit dysregulated glutamatergic signaling. Mn toxicity may impair glutamate clearance, disrupt glutamatergic homeostasis, and increase susceptibility to seizures. Indeed, in a recent study, using APP/PSEN1 and wild-type mice, researchers combined in vitro and in vivo approaches to assess astrocytic glutamate clearance and behavioral outcomes following acute Mn exposure. Results show that even limited Mn exposure accumulates in brain tissue, disrupts glutamate clearance in cortical astrocytes, and heightens seizure susceptibility without affecting hippocampal long-term potentiation (LTP). These effects occur in young adult mice before significant AD-related pathology, suggesting glutamate dysregulation as an early AD feature and Mn exposure as a potential amplifier of genetic risk [40].

#### 3.2.8. Astrocytic REST (Repressor Element-1 Silencing Transcription Factor) Role in Mn-Induced Neurotoxicity

Manganese (Mn) accumulates in the brain, especially in brain regions such as the striatum and globus pallidus, through disturbing mitochondrial function, oxidative stress, inflammation, and excitotoxicity—common neurodegenerative pathways observed in Alzheimer’s disease (AD) and Parkinson’s disease (PD). REST, a zinc-finger transcription factor, regulates neuronal and astrocytic gene expression, including glutamate transporters like EAAT2, which protect neurons from excitotoxicity. While neuronal REST’s protective role in AD and PD is well documented, the role of astrocytic REST remains underexplored. Studies show that astrocytic REST deletion exacerbates PD-related dopaminergic neuronal loss and increases inflammation. In Mn-exposed mice, REST deletion in the striatum led to worsened dopaminergic dysfunction, motor deficits, cognitive impairments, inflammation, and reduced glutamate transporter GLT-1. These findings highlight REST’s critical role in mitigating Mn-induced neurotoxicity and glutamate dysregulation, emphasizing astrocytic REST as a potential therapeutic target for Mn-related neurodegeneration and broader neurodegenerative disorders. The striatum was chosen for this study due to its vulnerability to Mn accumulation, excitotoxic lesions, and dopaminergic degeneration, providing insights into region-specific astrocytic REST functions [83].

#### 3.2.9. Manganese-Induced Dysregulation Amyloid Precursor Protein (APP) Processing and Cognitive Impairment

Excessive manganese (Mn) exposure causes cognitive deficits similar to Alzheimer’s disease (AD) by affecting amyloid precursor protein (APP) and its processing. APP, critical in AD pathology, undergoes cleavage by α- and β-secretases, influencing synaptic function. In Mn-exposed mice and Neuro-2a cells, APP, α-secretase (ADAM10), and soluble APP alpha (sAPPα) levels decreased, along with synaptic protein expression and α-secretase activity. However, β-secretase, Aβ peptides, and β-secretase activity were unaffected. Mn exposure alters non-amyloidogenic APP processing, impairing cognition and synaptic function, linking APP dysregulation to cognitive decline [84].

**Table 2:** Literature summary of the mechanistic studies for manganese (Mn) exposure and AD pathobiology.

SL	References	Study Objective	Study Type	Exposure	Key Findings
1	Fan et al. 2024 [82]	Investigated the role of Drp1 in Mn-exposure-induced autophagy and mitochondrial function. Determined if Drp1 inhibition improves autophagy independent of mitochondria.	HeLa cells, N27 neuronal cells and mice	Cells and mice—oral gavage (for cell: 62.5μM to 2mM for 24 or 48 h; for mice: ~4.2 mg absolute Mn/kg/day for 30 days)	A partial Drp1 loss of function appears to be safe and sufficient to confer neuroprotection against Mn-induced autophagy flux.
2	Spitznagel et al., 2023 [40]	Examined acute Mn exposure effect on glutamatergic neurotransmission, astrocytic glutamate clearance, behavior in AD models, and pre-symptomatic AD vulnerabilities.	Mice and primary astrocytes	Mice and primary astrocytes—injections (13.8 mg/kg for 1 week (injected on days 1, 4, and 7))	Mn exposure disrupts glutamate clearance, elevates GLAST (Glutamate Aspartate Transporter), and increases seizure susceptibility in APP/PSEN1 mice, potentially aiding early AD.
3	Pajarillo et al., 2022 [83]	Investigated astrocytic REST’s role in Mn-induced neurotoxicity, assessed locomotor and cognitive function impairment, and evaluated the impact of astrocytic REST deletion on proinflammatory factors.	Mice and primary astrocytes	Mice and primary astrocytes—nostril (30 mg/kg, 1 μL per nostril in both nostrils, daily for 21 days)	Astrocytic REST (Repressor Element-1 Silencing Transcription Factor) deletion worsens Mn toxicity, causing dopamine dysfunction, motor deficits, cognitive decline, and inflammation.
4	Xu et al., 2021 [78]	Investigated Mn-induced autophagy dysfunction in N2a cells, PP2Ac methylation in autophagy regulation, the effects of ABL-127 on PP2Ac methylation, and the impact of LCMT1 overexpression on autophagy.	N2a cells	N2a cells (250, 500, 1000, 2000 μM for 6, 12, and 24 h)	Mn cytotoxicity disrupts autophagy in neuronal N2a cells, a hall mark of AD. Regulating PP2Ac methylation may help prevent Mn neurotoxicity and neurodegenerative diseases like AD.
5	Rizor et al., 2021 [75]	Investigated Mn-induced YY1 activation via the NF-kB pathway and mechanisms impairing EAAT2 function in astrocytes.	H4 human astrocyte cells	Cells (250 μM, 3 h)	Mn exposure triggers oxidative stress, TNF-α production, IKK-β activation, YY1 upregulation, and EAAT2 repression.
6	Yang et al., 2021 [84]	Investigated Mn-induced cognitive impairment mechanisms by assessing the role of APP in cognitive deficits, APP’s secretase processing in neurotoxicity, and synaptic dysfunction by using both in vivo mouse model and in vitro cell culture (N2a cells).	Astrocyte cell	Mice and N2a cells—gastric gavage (25, 50, or 100 mg/kg for 90 days)	Mn exposure impairs cognition in mouse models and inhibits APP expression and α-secretase activity.
7	Pajarillo et al., 2020 [79]	Investigated the role of RE1-silencing transcription factor (REST) in dopaminergic neurons against Mn-induced toxicity and the enhancement of the expression of the dopamine-synthesizing enzyme tyrosine hydroxylase (TH).	Neuronal cell lines (Mouse CAD cell line and LUHMES (CRL-2927) cell line)	Cells (50, 100, 250 μM for 3, 6, 12 h)	REST enhances TH expression, protects dopaminergic neurons from Mn toxicity, reduces oxidative stress, regulates apoptosis, promotes antioxidants, and its dysfunction links to Parkinson’s and Alzheimer’s disease.
8	Lu et al., 2018 [80]	Investigated the impact of Mn-induced toxicity on the function of the gap junctional intercellular communication (GJIC) by examining the Cx43 expression, excitotoxicity cell death mechanisms, and glutamate homeostasis disruption.	Primary astrocytes	Primary astrocytes (125, 250, 500, 1000 μM for 4 and 16 h)	Mn exposure impairs astrocyte viability, increased apoptosis, disrupts glutamate homeostasis, increases intracellular glutamate levels, and downregulates glutamate transporter expression.
9	Kirkley et al., 2017 [81]	Investigated the role of microglia and glial crosstalk in Mn-induced neurodegeneration.	Mixed glial cultures from whole brain (astrocytes and microglia)	Cells (10, 30, 100 μM for 2, 4, 6, 8, 12, 24 h)	NF-κB signaling in microglia plays an essential role in inflammatory responses to Mn toxicity by regulating cytokines and chemokines that amplify the activation of astrocytes.

### 3.3. Mechanisms and Pathobiology of Alzheimer’s Disease Development Related to Lead Exposure

Lead (Pb) exposure-related toxicity was mentioned surprisingly as early as in 370 BC with major sources including old plumbing, paints in the old house, industrial applications, Pb smelters, waste incinerators, and automobile Pb-acid batteries [85]. Approximately 1% of global disease burden is associated with Pd exposure, which also has lasting effects on behavioral issues and children’s IQ [86,87]. Although Pb exposure is particularly more devastating on child health and development, it also contributes to various disease development in older adults including amyotrophic lateral sclerosis (ALS), Parkinson’s disease (PD), hearing loss, age-related cataracts, glaucoma, and other chronic conditions [85]. Environmental Pb exposure can occur through ingestion, inhalation, and endogenous sources, with the majority of exposure happening via inhalation or ingestion. Once in the body, Pb is absorbed into cells and tissues, circulating in the bloodstream. Pb can cross both the placental and blood–brain barrier (BBB), accumulating in the brain due to a low excretion rate. Pb, a well-established neurotoxicant, causes oxidative stress by depleting thiols and disrupting the antioxidant defense system. The primary neurotoxic mechanisms of Pb exposure include excessive oxidative stress, triggering endoplasmic reticulum stress, mitochondrial disfunction, and neuronal cell death; it also creates neuroinflammations, excitotoxicity, and disruption of the essential metal homeostasis in the brain [85].

Animal models (i.e., mouse, rats, and zebrafish) treated with Pb exhibit the mechanisms and symptoms of AD. While consistent AD-related deficits are documented, the effects of Pb exposure differ by species, timing, dose, and length of exposure. The current review discusses twenty-one mechanistic studies on Pb exposure and AD pathobiology. Among these, seven studies employed different cell lines, ten used mice as models and/or different cell lines, two used zebrafish, and two used rats as the model animal to investigate the link between environmental Pb exposure and AD pathobiology. Table 3 summarizes the main findings of these twenty-one mechanistic investigations and are discussed below.

#### 3.3.1. Lead Exposure Increases Tau Hyperphosphorylation, DNA Methylation, Amyloid Accumulation, Mitochondrial Dysfunction, Microglia Activation, and Neuronal Cell Death

Mice serve as the predominant animal model for investigating the effects of Pb exposure on the brain, owing to the availability of transgenic mice predisposed to AD. A study by Wang et al. (2022) demonstrated that miR-124-3p/BACE1 pathway modulation is critically involved in Pb-induced AD-like amyloidogenic processing in Pb-exposed mice [88]. Pb exposure contributes to the development of AD through several mechanism the in experimental mouse model, including the elevation of tau protein and mRNA levels in aged mice [89], reprogramming the expression of epigenetic intermediates involved in DNA methylation or histone modification that in turn regulate latent AD-related gene expression [90], early-life microglial damage and amyloid accumulation [91], changes in miRNA expression targeting AD-related proteins [92], elevated levels of phosphorylated tau protein (p-tau), abnormal changes in BBB junction proteins, and imbalanced production and clearance abilities of Aβ [93]. Pb exposure also increases permeability surface area product, affects brain perfusion and damages the BBB system [94], induces histone modification and DNA methylation [95], increases α-Synuclein, GSK-3β, Caspase-3, and tau hyperphosphorylation [96], and causes mitochondrial copper overload due to COX17-mediated translocation, mitochondrial dysfunction, excessive mtROS accumulation, and microglia activation [97].

#### 3.3.2. Lead Exposure Increases DNA Damage, Causes Excessive mtROS Accumulation, and Alters Calcium and Iron Homeostasis and Apoptosis

Researchers use several specific cell lines (i.e., SH-SY5Y, iPSCs, BV-2 microglial cells) in laboratory studies to model the AD pathology, test potential treatments, and investigate the role of amyloid beta plaques, tau protein abnormalities, and other key features of AD in a controlled environment. These cell experiments are essential to understand the molecular mechanisms at the cellular level. In this review, we highlight seven studies that used different cell lines to investigate the AD pathobiology and molecular mechanism due to environmental Pb exposure. The reported molecular mechanisms of Pb exposure-related AD are inhibited APP translation and disruption of iron homeostasis [98], PINK1/Parkin-mediated mitophagy and dysfunctional mitochondria-mediated apoptosis [99], altered calcium homeostasis, synaptic plasticity, elevated AD-like pathogenesis markers, including phosphorylated tau, tau aggregates, and Aβ42/40 [100], mitochondrial dysfunction and oxidative stress [101], mitochondrial dysfunction and excessive mtROS accumulation [97], oxidative stress and apoptosis [102], oxidative stress-mediated DNA damage, and a decrease in total antioxidant capacity and involvement of CDK5-p25 signaling [103].

#### 3.3.3. Lead Exposure Disrupts Brain Cholesterol Metabolism, Increases Aβ Accumulation and Amyloid Plaque Deposition, and Impairs Cholesterol Homeostasis

The laboratory rat has been a valuable model for brain research in disease models over the years. This review identifies two studies that examined the pathobiology of AD in relation to environmental Pb exposure using rat models. Zhou et al. (2018) demonstrated that Pb exposure contributed to early AD-like pathology in young growing rats by disturbing brain cholesterol metabolism and increasing Aβ accumulation and amyloid plaque deposition [44]. Pb exposure impaired cholesterol homeostasis, decreased brain cholesterol levels, activated the SREBP2-BACE1 pathway, decreased HMG-CR and LDL-R expression, and promoted the expression of LXR-α and ABCA1 [44]. In contrast, Liu et al. (2023) showed that chronic Pb exposure increased the affinity of Aβ40 to cerebral vasculature, intensified Aβ40 buildup, and impaired LRP1 expression in both the brain parenchyma and vasculature [104].

#### 3.3.4. Lead Exposure Disturbed Global Gene Expression Patterns in a Sex-Specific Manner

The zebrafish model shows potential for studying neurodegenerative disease mechanisms like AD. Embryonic exposure to Pb at levels as low as 10 µg/L disturbed global gene expression patterns in a sex-specific manner, leading to neurological alterations later in life. Pb exposure during embryogenesis in zebrafish led to neurodegenerative gene expression changes [105]. In a study examining the 12-month-old adult female and male zebrafish brain, exposed to either a control (0 μg/L) or 10 μg/L Pb only during embryogenesis (1–72 h post-fertilization), gene ontology and pathway analysis demonstrated that both sexes had similar upper disease and functional categories, but female zebrafish exhibited 4.3 times more genetic alterations. Genes linked to the development and function of the nervous system were particularly more affected; adult females were found to have altered versions of 89 genes linked to AD, including sortlin-related receptor precursor (SORL1), apolipoprotein (APOE), and amyloid precursor protein (APP) [105]. Another study showed that environmental Pb exposure causes de novo CNAs, which may be a mechanism causing neurological diseases. These CNAs are linked to AD outcomes with most genes are interconnected within a molecular network with amyloid precursor protein (APP), a crucial molecular target linked to the etiology of AD [106].

**Table 3:** Literature summary of the mechanistic studies for lead (Pb) exposure and AD pathobiology.

SL	References	Study Objective	Study Type	Exposure	Key Findings
1	Rogers et al., 2016 [98]	Investigated the effect of Pb on iron homeostasis proteins in human neurons.	Human neuroblastoma SH-SY5Y cells—in vitro	Cells (100, 250, 500, 750, 1000 µM for 24 h)	Pb inhibits APP translation, raising cytosolic iron levels. Through the restoration of APP production, iron supplementation protects cells from Pb toxicity.
2	Wang et al., 2022 [88]	Investigated how Pb affected microRNAs (miRNAs), post-transcriptional regulators that may be involved in the pathophysiology of AD.	Mice—animal model	Mice—drinking water (0.2% Pb acetate solution for 3 months)	Pb exposure modulates miR-124-3p/BACE1 pathway, upregulating BACE1 and impacting amyloidogenic processing resembling AD.
3	Bandaru et al., 2022 [99]	Investigated the mitophagy marker proteins, including PINK1 and Parkin, in differentiated SH-SY5Y cells to examine the impact of Pb exposure on the PINK1/Parkin dependent pathway.	SH-SY5Y cells	Cells (5 µM for 24 h)	Pb exposure decreases mitochondrial mass, elevates MPTP opening, depolarizes membrane potential, and increases ROS generation, inducing neurotoxicity through the PINK1/Parkin-mediated mitophagy.
4	Eid et al., 2016 [90]	Investigated how early life exposure to Pb can cause epigenetic modifications and late-life changes.	Mice model	Mice—drinking water (0.2% Pb-acetate from PND 1 to PND 20)	Prenatal Pb exposure alters epigenetic regulators, modifying AD-related gene expression via histone/DNA methylation changes.
5	Xie et al., 2023 [100]	Investigated the effects of Pb exposure on AD-like pathogenesis in human cortical neurons.	Human iPSC-derived cortical neurons as a model system	Neurons (15, 50 ppb for 25, 45 days)	Pb exposure disrupts Ca regulation, induces epigenetic changes, and promotes AD-related tau and Aβ pathology.
6	vonderEmbse et al., 2017 [91]	Investigated the association between early toxicant exposure and systematic microglia activation, possibly reversing the pathological severity of AD.	Mouse model	Mouse—gavage technique (100 ppm for 3–6 months)	Pb exposure activates microglia and increases amyloid buildup. Females might be more vulnerable to AD as a result of early-life microglial injury.
7	Masoud et al., 2016 [92]	Investigated the early postnatal Pb exposure and its effect on the expression of select miRNAs, targeting AD-related protein.	Mice model	Mouse—mother’s milk (0.2% Pb acetate from Postnatal Day 1 (PND 1, first 24 h after birth) to PND 20)	Pb exposure triggers changes in miRNA expression targeting AD-related proteins.
8	Lee and Freeman, 2016 [105]	Investigated the connection between latent neurological changes and embryonic Pb exposure utilizing the brains of adult male and female zebrafish.	Zebrafish brain	Aquaria water (10 µg/L Pb [~0.048 µM] until 12 months of age)	Pb exposure in zebrafish leads to neurodegenerative gene expression changes in a sex-specific manner.
9	Wu et al., 2020 [93]	Examined how Pb exposure exacerbates the development of AD in mice by compromising the blood–brain barrier (BBB).	Mice model	Mice—drinking water (200 mg/L and 500 mg/L Pb acetate, until the age of 7-months)	Pb exposure results in aberrant alterations in BBB junction proteins and hastens the deposition of Aβ1–42 in the brains of APP/PS1 mice including rise of p-tau expression in APP/PS1 and C57BL/6J mice.
10	Gu et al., 2020, [94]	Investigated long-term Pb exposure effect on the BBB’s permeability using the Dynamic Contrast-Enhanced Computerized Tomography (DCE-CT) technique.	Mice model	Mice—oral gavage (13.5 or 27 mg/kg Pb) for 4 weeks)	Pb exposure increases the permeability surface area product, significantly induces brain perfusion, disrupts the brain vasculature and damages the BBB system.
11	Bandaru et al., 2023 [101]	Examined how Pb induces AD, via mitochondrial damage, using human neuronal cells.	Human neuronal cells (SH-SY5Y)	Human neuronal cells (5 µM for 24 h)	Pb exposure increases oxidative stress, reduces GSH levels, and antioxidant-related gene expressions, such as SOD2 (MnSOD) and Gpx4; mitochondrial dysfunction resembling features of AD.
12	Lee and Freeman, 2020 [106]	Investigated the relation between neurotoxic Pb exposure and de novo copy number alterations (CNAs) using zebrafish fibroblast cells.	Zebrafish cells	Cells (0.24, 2.4, or 24 μM Pb for 72 h)	Pb exposure causes de novo CNAs, which may be a mechanism causing neurological diseases. Amyloid precursor protein (APP), a crucial molecular target linked to the AD pathophysiology is connected to nearly every gene in a molecular network.
13	Liu et al., 2023 [104]	Investigated long-term Pb exposure effect on Aβ buildup in cerebral capillaries and the expression of a vital Aβ transporter, low-density lipoprotein receptor protein-1 (LRP1), in brain parenchyma and capillaries in Sprague-Dawley rats.	Rat model	Rat—oral gavage (14, 27 mg/kg for 4 or 8 weeks.)	Pb exposure increases Aβ buildup in the cerebral vasculature both in vitro and in vivo, including reduction of LRP1 expression in the studied brain region and fractions.
14	Eid et al., 2018 [95]	Investigated early life Pb exposure and latent over expression of AD-related gene regulation histone activation pathways.	Mice model	Drinking water (0.2% Pb acetate from PND 1 to PND 20)	Pb exposure produces a global gene repression profile via DNA methylation and histone modification pathways except in the genes linked to AD.
15	Bihaqi et al., 2014 [89]	Investigated infantile postnatal Pb exposure on the expression of tau in the aged mice’s brain.	Mice model	Drinking water (0.2% Pb acetate from PND 1 to PND 20)	Pb exposure increases tau protein and tau mRNA levels, serine/threonine phosphatase activity, and a changed p35/p25 protein ratio.
16	Bihaqi et al., 2018 [96]	Examined developmental Pb exposure effects on α-Syn pathways in tau-knockout mice and SHSY5Y cells.	Mice and SHSY5Y cells	Drinking water (0.2% Pb acetate from PND 1 to PND 20 and 5, 50, 100 µM for 48 h for cells)	Pb exposure upregulates α-Syn, Caspase-3, glycogen synthase kinase 3β (GSK-3β), and α-Syn and its phosphorylated forms via epigenetic mechanisms.
17	Huang et al., 2024 [97]	Investigated how Pb exposure aggravates AD progression and the role of microglial activation using APP/PS1 mice and Aβ1-42-treated BV-2 cells.	Mice model and BV-2 microglial cells	Drinking water (100 ppm until 4 months of age)	Chronic Pb exposure exacerbates memory and learning deficits in APP/PS1 mice, activating microglia via the mitochondrial dysfunction and excessive mtROS accumulation.
18	Zhou et al., 2018 [44]	Investigated the role of cholesterol metabolism in Pb-induced premature AD-like pathology in rats.	Male Sprague-Dawley rats	Drinking water (0.5–2% Pb acetate for 4 weeks)	Pb exposure disrupts brain cholesterol metabolism, triggering SREBP2-BACE1, reducing HMG-CR and LDL-R, and increasing ABCA1 and LXR-α, causing AD-like pathology.
19	Ayyalasomayajula et al., 2019 [102]	Examined epigallocatechin gallate’s (EGCG) role in reducing oxidative stress, apoptosis in neural cells exposed to Pb, β-APs.	SH-SY5Y cells	Cells (5 μM for 24 h)	Pb exposure increases oxidative stress, annexin V, Caspase-3, and apoptosis through the alteration of expression levels of Bax and Bcl2.
20	Lee et al., 2017 [107]	Investigated embryonic Pb exposure effect on AD related genes via sex-specific alterations in sorl1 expression in adult zebrafish.	Zebrafish	Aquaria water (100 ppb for 72 hpf)	Females are more prone to the onset of AD and SORL1, an AD genetic risk factor plays a critical role for this phenomenon.
21	Lokesh et al., 2024 [103]	Examined Pb and Aβ peptides’ (1–40 and 25–35) interaction with CDK5/p25 to understand Pb-induced neurotoxicity in neurons.	Human SH-SY5Y cells	Cells (5 μM for 24 and 48 h)	Pb exposure alters calcium levels, reduces antioxidants, increases oxidative damage, and disrupts CDK5-p25 signaling, affecting DNA repair and metabolism.

### 3.4. Mechanisms and Pathobiology of Alzheimer’s Disease Development Related to Cadmium Exposure

Cadmium (Cd) is a naturally occurring heavy metal that is bluish white in color and persistent in nature. The earth’s crust serves as one of the natural sources of Cd, while anthropogenic sources include mining, refining, fossil fuel combustion, waste incineration, and other industrial activities such as manufacturing phosphate fertilizers [108]. Cd is not an essential element; therefore, it has no physiological function in humans. However, according to the International Agency for Research on Cancer (IARC), Cd is classified as a Group-I carcinogen (cancer causing agent) and has numerous detrimental health effects upon exposure [109]. Most cases of human exposure to environmental Cd are through contaminated food, making diet the primary source of Cd exposure [110]. Additionally, people are exposed to Cd through inhalation, particularly by inhaling cigarette smoke which is another significant route of exposure. Chronic environmental Cd exposure increases the risks of various human diseases including kidney damage, high blood pressure, diabetes, decreased pulmonary function, and osteoporosis [110]. Recently, Cd has also emerged as a neurotoxicant. Inhaled Cd can enter the brain through the olfactory bulb and the blood–cerebrospinal fluid barrier. Mechanistic studies have demonstrated that Cd exposure triggers oxidative stress, alters the permeability of the BBB, causes Aβ aggregation, produces tau neurofibrillary tangles, induces neuroinflammation, and leads to apoptotic neuronal cell death [85].

This review delineates nine mechanistic laboratory studies concerning environmental Cd exposure and AD pathobiology. Among these nine studies, two utilized cell lines (SN56 and Neuro-2a cells), six employed mouse models, and one used a rat model to examine the association between environmental Cd exposure and AD pathobiology. Table 4 presents the primary findings and details of these nine mechanistic studies regarding the influence of Cd exposure on the pathways of AD development. Mouse models and cell cultures treated with Cd exhibit mechanisms and symptoms of AD. However, the degree of influence of Cd exposure depends on several factors, including species, timing, dose, and length of exposure.

#### 3.4.1. Cadmium Exposure Increased Anxiety-like Behavior, Spatial Reference Memory Damage, Aβ Plaque Deposition, and Microglial Activation

Mouse models have shown promise in the neurological research field and providing insights into the molecular mechanisms underlying environmental Cd exposure and AD pathobiology. While oxidative stress, neuroinflammation, and apoptotic neuronal cell death are well-established molecular pathways involved in Cd exposure and AD pathobiology, emerging mechanisms continue to be identified over time. This current review identifies six mouse model studies related to environmental Cd exposure and its contribution in AD pathways. Among these studies, one explored the effects of low-dose environmental Cd exposure on AD progression and underlying mechanisms using wild-type C57BL/6J and APP/PS1 double transgenic mice. The findings are noteworthy as both genotypics (C57BL/6J and APP/PS1) exhibited similar Cd levels in the blood after exposure to the same Cd dose, yet the toxic effects differed in genotypes. Environmental Cd exposure exerts more severe damage in APP/PS1 mice compared to C57BL/6J mice, leading to increased anxiety-like behavior, chaotic movement, spatial reference memory damage, Aβ plaque deposition, and microglial activation in the brain, as well as increased expression of IL-6 in the cortex and serum. These results indicate that low-dose environmental Cd exposure contributes to AD progression through mechanisms, including BBB disruption, increased Aβ production, reduced Aβ clearance and increased inflammatory responses [111]. Other potential molecular mechanisms of Cd exposure-induced AD progression include genotype- and gender-specific gut dysbiosis, increased microbial AD biomarkers, reduced energy supply-related pathways in gut and blood, and enhanced hepatic pathways involved in inflammation and xenobiotic biotransformation [43].

In another study, Wang et al. (2022) demonstrated that Cd dysregulated brain and liver drug-processing genes in a sex- and ApoE-genotype-specific manner, contributing to variations in susceptibility to Cd neurotoxicity. Proinflammatory genes were enriched in Cd-exposed ApoE4 males’ livers, while Cd upregulated Cyp2j isoforms in the brains of ApoE3 mice. Dysregulation of cation transporters was male-specific in the brains [112]. Matsushita and colleagues investigated the genetic and conditional stimulation of adult neurogenesis and its rescue role in Cd-induced cognitive impairment in ApoE4-KI mice [113]. They showed that specific and conditional stimulation of adult neurogenesis rescued Cd-induced impairments in hippocampus-dependent short-term spatial memory in a gene–environment interactions (GxE) model of ApoE4 and Cd exposure, demonstrating a direct link between memory impairment and adult neurogenesis in the GxE mouse model [113].

Similarly, Zhang et al. (2020) revealed that a GxE between ApoE4 and Cd exposure accelerates cognitive impairment with impaired adult hippocampal neurogenesis identified as the potential mechanism. Furthermore, male mice were more susceptible than female mice to the GxE effect during youth [114]. Qian et al. (2024) reported that Cd promotes neural senescence by activating the p53/p21/Rb pathway. Their findings revealed that SigmaR1 depletion contributed to neural senescence through Ca^2+^ dyshomeostasis and mitochondrial dysfunction [115]. This mouse model, combined with SHSY5Y cell culture studies, revealed a novel senescence-associated regulatory route for the SEL1L/HRD1/SigmaR1 axis that affects the pathological progression of Cd exposure-associated AD. The study concluded that SigmaR1 functions as a neuroprotective biomarker of neuronal senescence and pharmacological activation of SigmaR1 could serve as a promising therapeutic strategy for AD [115].

#### 3.4.2. Cadmium Exposure Induces Neuronal Cell Death Through Apoptosis and Autophagy

Cell culture studies are crucial for elucidating the molecular mechanism and cellular pathways associated with environmental risk factors and AD pathobiology. Several cell lines, including the AD Pathophysiology Model (i.e., induced pluripotent stem cells (iPSCs), SH-SY5Y, PC12, and primary neuronal cultures), are commonly used as models of AD pathophysiology to study the molecular mechanism of AD progression and the contribution of the environmental factors. In this review, two in vitro studies using SN56 and Neuro-2a cells were identified for review and thoroughly discussed. Deng et al. (2024) carried out an in vitro study using mouse neuroblastoma cells (Neuro-2a cells) to investigate the effect of autophagy on environmental Cd-induced AD progression and the underlying molecular mechanisms [116]. Their findings revealed that Cd exposure disrupted autophagosome–lysosome fusion and impaired lysosomal function, leading to defects in autophagic clearance followed by APP accumulation and nerve cell death [116]. Their study also demonstrated that SIRT5 is an essential molecular target in Cd-impaired autophagic flux. Mechanistically, Cd exposure reduced SIRT5 expression, increasing the succinylation of RAB7A at lysine 31 and inhibiting RAB7A activity, which contributed to autophagic flux blockade. This indicates that SIRT5-catalysed RAB7A desuccinylation is an essential adaptive mechanism for the amelioration of Cd-induced autophagic flux blockade and AD-like pathogenesis [116]. Del Pino et al. (2016) investigated the mechanism of Cd exposure-induced basal forebrain cholinergic neuron cell death. They showed that Cd induces cell death on cholinergic neurons through the blockade of the M1 receptor, overexpression of AChE-S and GSK-3β, downregulation of AChE-R, and increase in Aβ and total and phosphorylated tau protein levels [117].

Arab et al. (2023) investigated the Cd-induced neurotoxicity mechanisms and examined the potential neuroprotective role of topiramate against Cd-induced cognitive deficits in rats, with an emphasis on hippocampal oxidative insult, apoptosis, and autophagy. They found that Cd exposure triggered spatial learning/retention memory impairments, deterioration of recognition memory, increased hippocampal neurodegeneration signals (elevated hippocampal levels of Aβ42 and p-tau), augmented hippocampal glutamate content, and altered the SIRT1/Nrf2/HO-1 axis and AMPK/mTOR signaling pathway [118].

**Table 4:** Literature summary of the mechanistic studies for cadmium (Cd) exposure and AD pathobiology.

SL	References	Study Objective	Study Type	Exposure	Key Findings
1	Liu, et al., 2023 [111]	Examined how low-dose environmental Cd exposure contributes to AD development.	Mice model	Mice—drinking water (1, or 10 mg/L for until 6 months of age)	Cd exposure causes a rise in anxiety-like behavior and disorderly movement, disruption to spatial reference memory, Aβ plaque formation in mice brains, an increase in microglia expression in the brain, and elevates IL-6 levels in the cortex and serum.
2	Zhang et al., 2021 [43]	Investigated Cd interactions with ApoE4 gene variants to modify the gut–liver axis in mice.	Mouse model	Mice—drinking water (0.6 and 3 mg/L for 14 weeks)	Cd exposure disrupts the gut–liver axis, increasing microbial AD biomarkers and inflammatory hepatic pathways.
3	Wang et al., 2022 [112]	Investigated Cd-ApoE effects on the transcriptome alterations in the livers and brains of ApoE3/ApoE4 transgenic mice.	Mice model	Mice—drinking water (0.6 mg/L for 6 weeks)	Cd dysregulates drug-processing genes in brain and liver, varying by sex and ApoE genotype.
4	Matsushita et al., 2023 [113]	Explored rescuing of Cd-induced cognitive impairment in ApoE4-KI mice via genetic and neurogenesis activation.	Mice model	Drinking water (0.6 mg/L)	Selective and conditional stimulation of adult neurogenesis restores Cd-induced impairments in hippocampus-dependent short-term spatial memory.
5	del Pino et al., 2016 [117]	Investigated how Cd induces cell death in basal forebrain cholinergic neurons.	SN56 Cell lines	SN56 cells (1, 10, 100 µM for 24 h)	Cd exposure induces cell death in cholinergic neurons, by blocking the M1 receptor, overexpressing AChE-S and GSK-3β, downregulating AChE-R, and raising the levels of Aβ, total, and phosphorylated tau proteins.
6	Deng et al., 2024 [116]	Investigated the underlying mechanism and impact of autophagy on the development of AD caused by environmental Cd.	Mouse neuroblastoma cells (Neuro-2a cells)	Cells (1, 2, and 4 μM for 24, 48 and 72 h)	Cd exposure disrupts autophagy, causing APP buildup and neuronal death.
7	Arab et al., 2023 [118]	Examined topiramate’s potential to counter Cd-induced cognitive deficits via hippocampal oxidative stress, apoptosis, and autophagy.	Rats model	Rat—oral gavage (5 mg/kg/day for eight weeks)	Cd exposure impairs retention memory, deteriorates the recognition memory, triggers hippocampal neuronal degeneration and signals as well as induces hippocampal apoptotic and autophagic cell death.
8	Zhang et al., 2020 [114]	Examined ApoE-e4 and Cd exposure interactions on cognition using an Alzheimer’s mouse model expressing human ApoE-e3 (ApoE3-KI [knock-in]) or ApoE-e4 (ApoE4-KI).	Mouse model	Mouse—drinking water (0.6 mg/L for 14 weeks)	Cd exposure accelerates cognitive decline, likely via reduced hippocampal neurogenesis.
9	Qian et al., 2024 [115]	Examined cellular senescence in AD by exploring Cd exposure effects on neuron senescence in vivo/vitro.	Mice and SHSY5Y cells	Mice—drinking water (10 mg/L for 7 weeks)	Cd induces neural senescence via p53/p21/Rb activation and SEL1L/HRD1-mediated SigmaR1 degradation, suggesting SigmaR1 as a neuroprotective biomarker.

## 4. Discussion

The growing body of research on environmental toxicants has highlighted the significant roles of arsenic (As), manganese (Mn), lead (Pb), and cadmium (Cd) in the development and progression of neurodegenerative diseases, particularly Alzheimer’s disease (AD). These toxicants induce a range of pathological processes that intersect with well-known mechanisms of AD, such as oxidative stress, mitochondrial dysfunction, protein aggregation, and neuroinflammation. The studies reviewed here provide a deeper understanding of how these elements contribute to AD and suggest potential pathways for therapeutic intervention.

As exposure has long been recognized for its carcinogenic properties, but emerging evidence has also linked it to neurodegenerative diseases. Chronic exposure to As in animal models results in mitochondrial dysfunction, tau hyperphosphorylation, and amyloid-beta (Aβ) accumulation—key features of AD pathology. These effects are coupled with disruptions in memory, circadian rhythms, and neuroinflammation, all of which are hallmarks of neurodegenerative conditions. Mechanistically, As has been shown to interact with critical protein kinases such as glycogen synthase kinase 3β (GSK3β) and extracellular signal-regulated kinase 1/2 (ERK1/2), leading to tau aggregation and the exacerbation of Aβ accumulation. These insights position As as an environmental toxicant that could significantly contribute to the development of AD in exposed populations.

**Figure 2:**
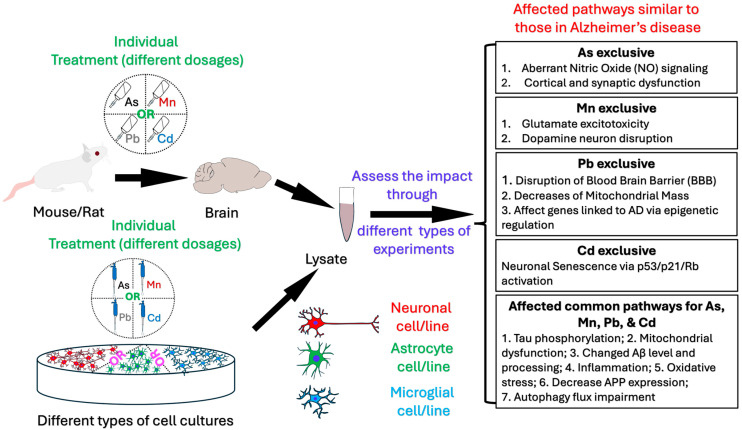
Summarized findings of discussed mechanistic studies. Using animal and cell line studies, it was observed that while exposure to individual metals (As, Mn, Pb, Cd) shares some affected pathways—such as tau phosphorylation, mitochondrial dysfunction, changed Aβ level and processing, inflammation, oxidative stress, decreased APP expression, and autophagy flux impairment—certain effects are unique to specific metals. For example, Pb disrupts the blood–brain barrier (BBB), decreases mitochondrial mass, and affects genes linked to AD via epigenetic regulation. Cd induces neuronal senescence via p53/p21/Rb activation. As causes aberrant NO signaling, as well as cortical and synaptic dysfunction, and Mn induces glutamate excitotoxicity and dopamine neuron disruption.

Similarly, Mn toxicity has been implicated in the pathogenesis of neurodegenerative diseases, particularly through its effects on glutamate regulation and neuroinflammation. Mn exposure leads to the suppression of the excitatory amino acid transporter 2 (EAAT-2), resulting in excitotoxicity and neuroinflammation. This dysregulation contributes to synaptic dysfunction, cognitive deficits, and abnormal amyloid precursor protein (APP) processing. The disruption of autophagy, an essential cellular process for clearing damaged proteins, further exacerbates the pathological effects of Mn. Research also suggests that transcription factors like the RE1-silencing transcription factor (REST) may play a protective role against Mn-induced neurotoxicity by modulating oxidative stress and inflammation, offering potential therapeutic strategies (Figure 2).

Pb and Cd, while historically associated with other toxicological concerns, have more recently emerged as significant neurotoxicants linked to AD. Pb exposure disrupts calcium homeostasis, synaptic plasticity, and mitochondrial function, processes that are integral to the development of neurodegenerative diseases. Pb’s ability to induce oxidative stress and epigenetic reprogramming further exacerbates its neurotoxic effects. On the other hand, Cd has been shown to activate the p53/p21/Rb pathway, inducing neural senescence, while also disrupting gut–brain interactions and aggravating neuroinflammation. Both Pb and Cd impair autophagic flux and mitochondrial function, leading to cellular dysfunction and contributing to AD pathology (Figure 2).

Employing a variety of models, including mice, rats, and zebrafish, the recent mechanistic studies have enhanced our understanding of how environmental toxins might contribute to the development and progression of AD. They also reveal how sex-specific differences and gene–environment interactions influence the molecular pathways through which environmental toxicants exert their effects. However, there is still much to learn, especially about the long-term effects of low-level toxicant exposure in real-life settings. Additionally, differences in genetic backgrounds and environmental variables across studies present challenges in drawing broadly applicable and generalizable conclusions.

While it is well recognized that humans are exposed to complex mixtures of metals, our review has specific emphasis on elucidating the mechanisms by which single-metal exposure may contribute to the pathogenesis of AD. This approach allows for a more precise understanding of the distinct neurotoxic effects of individual metals, which is essential for identifying specific pathways involved in AD development. By systematically evaluating studies from the last decade, we provide an updated synthesis of recent advancements in this field, ensuring that our discussion reflects the most current understanding of metal-induced neurodegeneration. However, we acknowledge that our specific focus inherently minimized the number of studies to be considered for this review, as a limited number of investigations addressing single-metal exposure has been found. Despite this limitation, our review offers critical insights into the molecular and cellular mechanisms underlying single metal-induced neurotoxicity, contributing to a more refined framework for future research. Furthermore, by emphasizing individual metal toxicity, our review serves as a foundational reference that can guide future studies on metal mixtures and their synergistic or antagonistic effects associated with AD pathobiology.

## 5. Conclusions and Future Directions

The evidence presented here clearly points to arsenic (As), manganese (Mn), lead (Pb), and cadmium (Cd) as critical environmental risk factors for the development and progression of Alzheimer’s disease (AD). These metals disrupt essential brain processes such as oxidative stress regulation, mitochondrial function, and autophagy, all of which contribute to neurodegeneration. The impact of As on tau phosphorylation and amyloid-beta accumulation, the role of Mn in glutamate dysregulation and excitotoxicity, and the effects of Pb and Cd on mitochondrial bioenergetics and cellular senescence highlight the intricate molecular mechanisms through which these toxicants contribute to AD.

While animal and cell line studies have provided invaluable insights into the molecular and cellular underpinnings of metal-induced neurotoxicity, further research is needed to address existing gaps. A more comprehensive understanding of the cumulative effects of chronic, low-level exposure to these toxicants, particularly in the context of genetic susceptibility, is essential for developing targeted therapeutic strategies. The complexity of Alzheimer’s disease necessitates a holistic approach that integrates environmental, genetic, and epigenetic factors.

### 5.1. Future Directions

Future research should prioritize several key areas to enhance our understanding of environmental contributions to AD and identify potential interventions.

#### 5.1.1. Longitudinal and Epidemiological Studies

Large-scale studies that assess the cumulative effects of As, Mn, Pb, and Cd exposure on neurodegeneration are essential. These studies should be designed to track exposure levels over time and examine their correlation with cognitive decline and AD incidence. Epidemiological data will provide critical insights into the dose-dependent effects of these metals and their role in the progression of AD in human populations.

#### 5.1.2. Mechanistic Studies

Further research is needed to explore the interaction between environmental exposures and genetic susceptibility to AD. For example, studies could investigate how genetic risk factors such as APOE4 interact with metal-induced oxidative stress, inflammation, and mitochondrial dysfunction. Understanding these interactions could reveal novel biomarkers for early diagnosis and targeted therapeutic interventions.

#### 5.1.3. Therapeutic Development

Targeting the molecular pathways affected by As, Mn, Pb, and Cd exposure could lead to the development of new therapeutic strategies for AD. Inhibitors of kinases like GSK3β and ERK1/2, which are involved in tau phosphorylation and aggregation, could be explored as potential treatments. Additionally, enhancing the function of transcription factors like REST, which may protect against metal-induced neurotoxicity, could offer a novel therapeutic approach.

#### 5.1.4. Intervention Studies

Investigating the protective effects of antioxidants, chelating agents, and epigenetic modulators may provide strategies to counteract the neurotoxic effects of these metals. Compounds that restore glutamate transporter function or enhance mitochondrial resilience could mitigate excitotoxicity and bioenergetic deficits, potentially providing therapeutic benefits.

#### 5.1.5. Environmental Policy and Public Health

Translating research findings into actionable policies is crucial for mitigating the impact of environmental toxicants on public health. Efforts to regulate As, Mn, Pb, and Cd levels in drinking water and food sources, coupled with public education campaigns, could reduce exposure and the associated risks of neurodegenerative diseases. Stronger environmental regulations and improved waste management practices are key to preventing further contamination.

#### 5.1.6. Advanced Models

The use of organoid models and humanized systems will improve the translational relevance of findings. These models can better replicate human-specific aspects of AD pathology and allow for high-throughput screening of potential interventions. By advancing animal models that more closely mimic human AD, we can enhance the ability to translate preclinical findings into clinical applications.

In conclusion, addressing environmental contributions to AD, particularly from As, Mn, Pb, and Cd exposure, is essential for mitigating the growing public health burden of neurodegenerative diseases. By integrating mechanistic studies, longitudinal research, and public health initiatives, we can improve our understanding of these environmental risk factors and develop more effective strategies for prevention and treatment.

## Data Availability

No new data were created or analyzed in this study.
